# To Repair a Broken Heart: Stem Cells in Ischemic Heart Disease

**DOI:** 10.3390/cimb46030141

**Published:** 2024-03-08

**Authors:** Theodora M. Stougiannou, Konstantinos C. Christodoulou, Ioannis Dimarakis, Dimitrios Mikroulis, Dimos Karangelis

**Affiliations:** 1 Department of Cardiothoracic Surgery, University General Hospital of Alexandroupolis, Dragana, 68100 Alexandroupolis, Greece; konstantinoschristodoulou@yahoo.gr (K.C.C.); dmikrou@med.duth.gr (D.M.); dimoskaragel@yahoo.gr (D.K.); 2 Division of Cardiothoracic Surgery, University of Washington Medical Center, Seattle, WA 98195, USA; ijd22@uw.edu

**Keywords:** cardiovascular disease, coronary artery disease, myocardial infarction, cardiac surgery, pluripotent stem cells, multipotent stem cells, tissue engineering, myocardial patch, cell sheets, cardiac regeneration, cardiomyocyte proliferation

## Abstract

Despite improvements in contemporary medical and surgical therapies, cardiovascular disease (CVD) remains a significant cause of worldwide morbidity and mortality; more specifically, ischemic heart disease (IHD) may affect individuals as young as 20 years old. Typically managed with guideline-directed medical therapy, interventional or surgical methods, the incurred cardiomyocyte loss is not always completely reversible; however, recent research into various stem cell (SC) populations has highlighted their potential for the treatment and perhaps regeneration of injured cardiac tissue, either directly through cellular replacement or indirectly through local paracrine effects. Different stem cell (SC) types have been employed in studies of infarcted myocardium, both in animal models of myocardial infarction (MI) as well as in clinical studies of MI patients, including embryonic stem cells (ESCs) and induced pluripotent stem cells (iPSCs), Muse cells, multipotent stem cells such as bone marrow-derived cells, mesenchymal stem cells (MSCs) and cardiac stem and progenitor cells (CSC/CPCs). These have been delivered as is, in the form of cell therapies, or have been used to generate tissue-engineered (TE) constructs with variable results. In this text, we sought to perform a narrative review of experimental and clinical studies employing various stem cells (SC) for the treatment of infarcted myocardium within the last two decades, with an emphasis on therapies administered through thoracic incision or through percutaneous coronary interventions (PCI), to elucidate possible mechanisms of action and therapeutic effects of such cell therapies when employed in a surgical or interventional manner.

## 1. Introduction

Cardiovascular disease (CVD) represents a group of disorders generally affecting cardiac and vascular tissue; recent data presented by the American Heart Association (AHA) have shown that in 2020, about 19 million deaths worldwide were attributed to CVD, a statistic that seems to be increasing compared to the previous decade (2010) by about 18.7% [1]. Ischemic heart disease (IHD) in particular, often described as the most common type of heart disease, affects almost 5% of individuals over the age of 20, while it seems to be responsible for 375,476 deaths in 2021 alone, based on data provided by the World Health Organization (WHO) [2]. In general, IHD refers to a group of complex disorders of inflammatory nature [3], resulting in vascular remodeling of the coronary vessels causing a variety of stenotic and non-stenotic lesions. Some of these lesions eventually lead to tissue ischemia or even infarction [4].

Lifestyle modifications, guideline-directed medical therapy, as well as interventional and surgical methods, are often employed to either treat, alleviate, or reverse the underlying obstruction, achieving revascularization and reperfusion [5,6,7,8]. Nevertheless, these procedures might be associated with complications [9,10,11]. Furthermore, the problem at hand might not be fully addressed with the first intervention, often requiring repeat procedures [12,13]; with re-operative cardiac surgery in particular, there might be increased morbidity and mortality [14]. An additional therapeutic measure could thus present an appealing solution to facilitate tissue healing and regeneration after myocardial infarction (MI).

It is therefore the aim of this narrative review to present and critically evaluate some advances in the use of stem cells (SC) for IHD, particularly with regard to therapies administered through local thoracotomy or through interventional methods, and through which myocardial injury and local cardiomyocyte loss might be tackled or even reversed.

## 2. Ischemic Heart Disease (IHD)

### 2.1. Pathophysiology of Ischemic Heart Disease (IHD)

Ischemic heart disease (IHD) may be described as a complex group of pathophysiological and clinical entities comprising both acute and chronic events, such as acute myocardial ischemia and infarction and chronic coronary disease [15]. Various mechanisms have been found to contribute to disease progression, including atherosclerosis, inflammation, coronary vessel reactivity and microvascular dysfunction [16] (Table 1).

Atherosclerosis may be identified as the main contributor to the progression of IHD, a progressive, chronic disease comprising the development of atherosclerotic plaques within arterial vessels, attributable to both genetic and environmental factors [17]. The inciting event is usually endothelial dysfunction, characterized by loss of nitric oxide (NO) expression and changes in endothelial adhesion and permeability [18], usually brought on by hyperlipidemia, hypertension, smoking, elevated homocysteine, or hemodynamic factors including flow and shear alterations [19]. Increased leukocyte adhesion, particularly of monocytes, contributes to inflammation, leading to the generation of foam cells via lipoprotein engulfment. There is also increased local secretion of factors, including platelet-derived signaling molecules, fibroblast growth factor (FGF), and transforming growth factor beta (TGF-β). Eventually, recruitment of vascular smooth muscle cells (VSMC) will occur, leading to secretion of extracellular matrix (ECM), while in later stages, there may also be calcification and accumulation of necrotic debris within a forming lipid core, with variable effects on plaque stability [20,21].

Inflammation is a prominent feature in the pathophysiology of IHD, both during the pathogenesis of the atherosclerotic plaque and during myocardial infarction (MI) [22]. In the early phases of atherogenesis, oxidized low-density lipoprotein (oxLDL) within the plaque may lead to destabilization and facilitate monocyte adhesion through lectin-like oxidized low-density lipoprotein receptor 1 (LOX-1). LOX-1, when activated by oxLDL, induces NF-kB (nuclear factor ‘kappa-light-chain-enhancer’ of activated B-cells, nuclear factor kappa B) and adhesion molecule expression in affected endothelial cells [23]. In turn, this aids in the adhesion of circulating monocytes, contributing to endothelial cell apoptosis [16].

Vasospasm may be another factor contributing to the presentation and progression of IHD, often in locations with significant atherosclerotic burden [24]. Vasospasm may occur throughout various stages of atherosclerosis [25], while clinically, it may manifest as variant or prinzmetal angina. In this case, the myocardial ischemia is unrelated to any change in myocardial oxygen demand that might occur [26].

Increased coronary vessel reactivity, a mechanism also shown to contribute to disease progression, usually depends on endothelial dysfunction, or high innate vascular reactivity. In the case of endothelial dysfunction, the production of vasodilator substances such as nitric oxide (NO) is disturbed, hindering vasodilation. Vasoactive substances, including acetylcholine, serotonin (5-HT) and histamine, may induce both vasodilation and vasoconstriction through varying effects on different targets, including endothelial cells and VSMCs, respectively. Furthermore, vasoconstriction does not seem to be caused solely by impaired nitric oxide (NO) production [27], since vasoconstriction after endothelial dysfunction and mechanical irritation of coronary arteries due to intracoronary catheterization has also been reported [28]. The main mechanism underlying innate coronary vessel reactivity, usually attributed to both environmental factors (smoking) and genetic polymorphisms [29], includes Rho kinase (ROCK) upregulation, which may increase myosin light chain (MLC) sensitivity to Ca^2+^, both directly and indirectly, through inhibition of myosin light chain phosphatase (MLCPh) [27]. ROCK has also been associated with the proper functioning of various cytoskeleton proteins, leukocyte adhesion, as well as arterial and pulmonary arterial hypertension [30,31,32]. Protein kinase C (PKC) may also contribute to the innate coronary reactivity mechanism through MLCPh inhibition [27].

Microvascular dysfunction also contributes to IHD pathology, especially in cases of hyperglycemia or impaired glucose tolerance. Advanced glycosylation end products (AGEs) and reactive oxygen species (ROS) impair the function of antioxidant systems and decrease nitric oxide (NO) bioavailability, eventually causing endothelial dysfunction. Vascular permeability is also increased through the diacylglycerol (DAG)-PKC signaling pathway [16]. Aging also contributes to microvascular dysfunction through increased ROS and reactive nitrogen species (RNS) production [33].

Eventually, overt clinical manifestations may occur [34,35,36]; though initially, plaque growth induces outward vascular remodeling, continued growth will eventually impinge on the vascular lumen, decreasing its cross-sectional area. In chronic stable angina, this usually manifests as chest pain during exertion and is not usually associated with tissue necrosis and cardiomyocyte death [4,37]. On the other hand, acute plaque changes due to plaque erosion or rupture, a sudden increase in plaque size, acute vascular occlusion due to intraplaque hematoma, or plaque ulceration exposing various plaque constituents may lead to thrombosis and acute coronary occlusion [38]. Clinically, this may present as unstable angina or myocardial infarction (MI) [39].

Prolonged obstruction in blood flow eventually enables a series of tissue and cardiomyocyte changes associated with myocardial ischemia, a cessation of aerobic metabolism (decrease in creatine phosphate and adenosine phosphate-ATP), and an increase in toxic metabolites (lactic acid). After 20 to 30 min, ischemia becomes irreversible and myocardial infarction (MI) occurs, characterized by irreversible cardiomyocyte loss along with additional cellular changes [40,41], as described below.

Cardiomyocyte loss may occur through necrosis, a form of cell death characterized by several changes, including cell membrane swelling, depletion of intracellular ATP, dysfunction of ionic transporters and intracellular Ca^2+^ accumulation. The resulting cardiomyocyte fragments are immunogenic, activating Toll-like receptors (TLR) and leading to NF-kB activation, further potentiating an immune response. Apoptosis may also occur, a programmed form of cell death activated by oxidative stress and proinflammatory cytokines, once again resulting in cellular fragmentation and the formation of apoptotic bodies [41]. Forms of cell death such as necroptosis have also been described, occurring mostly during ischemia-reperfusion injury, through tumor necrosis factor receptor 1 (TNFR1)-mediated activation of the receptor-interacting protein (RIP) necroptotic protein complex (RIP-RIP3) [42,43]. Cardiomyocyte fragments and other cell constituents act as danger-associated molecular signals (DAMPS), binding various pattern recognition receptors (PRR); eventually, activation of various protein kinases and NF-kB expression leads to the production of various pro-inflammatory cytokines (tumor necrosis factor alpha, TNF-α, interleukins-1β, 6 and 18, (IL-1β, IL-6, IL-18)) [44]. Eventually however, inflammatory, and cellular debris is cleared through the activation of specific macrophage populations, a process known as efferocytosis [45,46,47]. A fibrous, collagen-rich scar is formed at the site of injury after the propagation of pro-fibrotic and angiogenic signals, occurring in response to the secretion of interleukin-10 (IL-10), transforming growth factor-β (TGF-β), and vascular endothelial growth factor (VEGF) [48,49].

### 2.2. Current Treatment Strategies for Ischemic Heart Disease (IHD)

IHD may be tackled through lifestyle measures, including physical exercise (provided there are no contraindications), as well as healthy dietary measures. This approach may be further augmented through guideline-directed medical therapy, in which case treatment and approach may differ depending on disease type, i.e., whether chronic coronary disease (CCD) or any of the acute coronary syndromes [50] (unstable angina, ST-segment-elevation-STEMI, non-ST-segment-elevation-NSTEMI) syndromes [51]. Interventional revascularization may also be attempted through percutaneous coronary interventions (PCI) or cardiac surgery; surgery is often recommended for left main disease associated with increased disease complexity (measured with a SYNTAX-‘Synergy between PCI with taxus and cardiac surgery’ score greater than 33), left main-equivalent disease (lesion in the proximal left anterior descending artery-LAD), as well as complex multivessel disease (once again with a SYNTAX score greater than 33), among others. In diabetic patients, surgical revascularization is recommended in instances of multivessel disease (including left anterior descending disease, LAD), unless they are poor surgical candidates or have low disease complexity [52].

Despite a multitude of different treatment modalities, including various revascularization strategies [52], cardiomyocyte loss after myocardial infarction (MI) is often irreplaceable [53]. Furthermore, the procedures themselves might be associated with side effects, in the case of fibrinolysis, or complications, in the case of percutaneous revascularization procedures or cardiac surgery. For example, in percutaneous interventions, there may be coronary perforation, dissection of vascular structures, or even major hemodynamic complications [54]. There may also be adverse effects, such as, for example, a possible kidney injury related to the use of intravascular dye in these procedures [55]. In addition, complications may arise with surgical revascularization as well, including stroke, renal failure, prolonged intubation times, infection of the sternum and associated deep tissues, as well as re-operation [56]. Thus, limitations related to incomplete myocardium restitution and the possibility for moderate to severe complications have further fueled research of therapeutic means that may complement or improve current management strategies [56].

## 3. Stem and Progenitor Cell Therapies for Ischemic Heart Disease (IHD)

Despite observed instances of cardiac regeneration in other species [57], cardiac tissue in adult humans has been generally described as incapable of regeneration [58,59]. In zebrafish, where cardiac regeneration was described for the first time, complete heart tissue replenishment has been shown to occur even after 20% resection of ventricular tissue within 2 months, a capability that seems to be lacking in zebrafish with *Mps1 (Monopolar spindle 1*) mutations [60,61]. Cardiomyocyte proliferation commonly occurs during the embryonic period through an intricate interplay between various signaling pathways, including Notch (Neurogenic locus notch homolog), neuregulin, Hippo and Wnt (Wingless-related integration site)/Frizzled [62,63,64,65,66,67]. During the postnatal period, however, the main mechanism of cardiac tissue growth seems to be physiological hypertrophy, mainly mediated through insulin/insulin growth-factor (IGF) signaling, activating phosphoinositide-3-kinase (PI3K)/mammalian target of rapamycin (mTOR)/Serine-threonine kinase Akt or Protein kinase B (AKT) [68], and RAS (Rat sarcoma protein, RAS)/RAF (Rapidly accelerated fibrosarcoma, RAF)/Mitogen-activated protein kinase kinase (MEK)/Mitogen-activated protein kinase (MAPK) signaling cascades [69], as well as signaling activated through thyroid hormone receptor signaling [70].

Though postnatal cardiac growth is mostly hypertrophic, various forms of cardiac regeneration may be seen across mammal neonatal and adult hearts, diminishing after the seventh postnatal day in mice [71] and after the second day in swine [72]. In humans, there does seem to be some cardiomyocyte turnover, albeit minimal, at around 1% per year, with some researchers placing this number at about 0.45% to 2% per year [73,74]. This turnover seems to occur through various mechanisms, with the most common one comprising a process of dedifferentiation, proliferation and finally redifferentiation from pre-existing cardiomyocytes commonly observed in the margins of an infarct [75]. New cardiomyocytes may also be generated through differentiation from local cardiac progenitor/stem cell populations (CPC/CSC) [74,76,77,78], or after transplantation of other stem and progenitor cells through transdifferentiation or cell fusion [74,79].

In any case, the limited regenerative capability displayed by endogenous cardiomyocytes is not adequate to fully address the incurred cardiomyocyte loss [57,80,81,82]. Though revascularization may restore the flow of oxygen and nutrients to an injured area [13], local cardiomyocyte populations are indeed not completely restored, with infarcted areas often replaced by collagen-rich fibrous scars [83,84]. To tackle this problem, various studies have examined the effect of stem cells or stem cell-related products on infarcted myocardial tissue, both in a preclinical as well as a clinical setting [85].

### 3.1. Cell Therapies

Among the possible strategies employed to aid in cardiomyocyte regeneration after myocardial infarction (MI), local transplantation of stem or progenitor cell populations is one possible option. Different cell types may be utilized, while their effects and safety have been tested either with preclinical studies, clinical trials, or both.

Although several studies have been conducted thus far, detailed analysis and comparison of these is beyond the scope of this text; instead, some indicative studies shall be presented for each cell type, including studies where the therapy in question was administered in a manner that might favor its administration during an invasive or interventional cardiac procedure. This is to shed light on possible mechanisms of action and the general effects of such therapeutic interventions on ischemic and infarcted myocardial tissue post-MI.

#### 3.1.1. Pluripotent Stem Cells in Animal Studies and Clinical Trials

**Figure 1:**
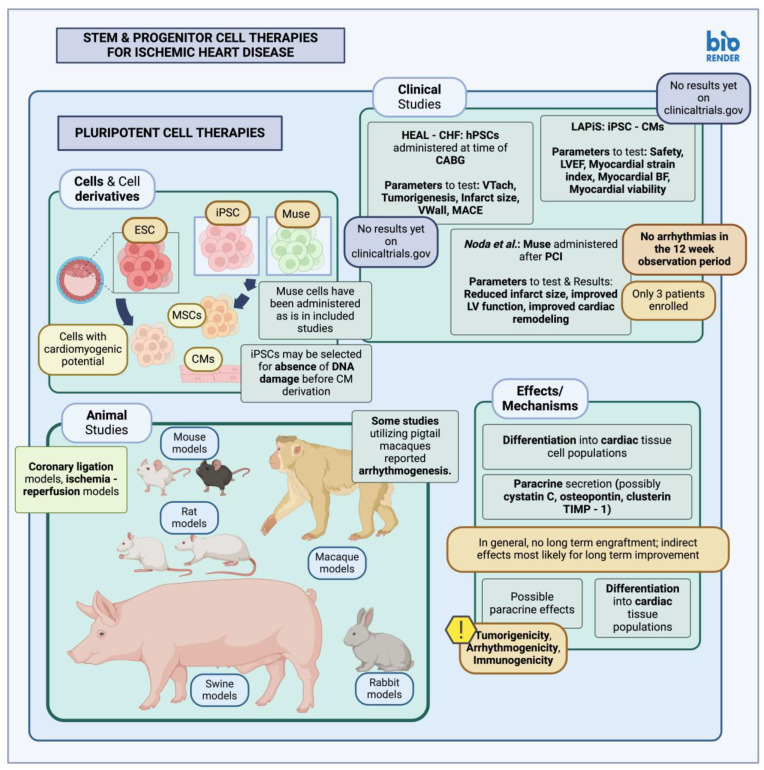
Stem and progenitor cell therapies for ischemic heart disease—pluripotent stem cell therapies (created with BioRender.com, accessed on 24 February 2024) [89,90,91]. CM, Cardiomyocyte; TIMP-1, tissue inhibitor of metalloproteinase 1; LV, left ventricle; BF, blood flow; VTach, ventricular tachycardia; VWall, ventricular wall; LVEF, left ventricular ejection fraction; iPSC, induced pluripotent stem cells; hPSC, human pluripotent stem cells; MSC, mesenchymal stem cells; ESCs, embryonic stem cells; CHF, congestive heart failure; CABG, coronary artery bypass graft; MACE, major adverse cardiovascular events; PCI, percutaneous coronary intervention.

Pluripotent stem cells (PSC), i.e., cells capable of generating tissue from all three germ layers that compose an adult organism [86], including induced PSCs (iPSCs) and embryonic stem cells (ESC), may be transplanted either directly or used for cardiomyocyte generation and subsequent administration. Several preclinical studies have tested the effect of PSC transplantation within infarcted myocardial tissue (summarized in Table 2, Figure 1). In particular, murine ESCs (mESCs) derived from mouse blastocysts have been transplanted into murine models of MI through local intramyocardial injection. These transplanted mESCs prevented cardiomyocyte hypertrophy and mitigated the local post-MI collagen deposition [87]. Another study by Min et al., testing the effect of directly transplanted ESCs, reported positive results with improved ventricular function due to paracrine and angiogenic effects, as well as the generation of differentiated cardiomyocyte progeny within the site of injury [88].

PSCs may also be used indirectly for the derivation of cardiomyocyte (CM) populations, or PSC-CMs, which can then be transplanted onto the target area [97]. Hatani and Yoshida have described protocols for the derivation and subsequent administration of hiPSC-derived cardiomyocytes in immunodeficient mice [98]. Animal studies using direct myocardial administration of stem cell populations from a starting PSC population, i.e., PSC-derived mesenchymal stem cells (MSC), have shown promising results with better efficacy compared to MSCs acquired from other locations [93]. Selecting PSC-derived cells with intact DNA structure has also facilitated the integration rate of transplanted cardiomyocytes onto target tissue [92]. Similar experiments have been carried out in non-human primates as well, with Kobayashi et al. describing protocols for the administration of PSC-derived cardiomyocytes in the *Cynomolgus* monkey after establishing a model of myocardial infarction [99]. Similarly executed experiments in the *Macaca nemestrina* species yielded promising results, including reduced infarct size and the generation of myocardial tissue in the injured area, although instances of ventricular arrhythmias were observed [95,99].

Direct transplantation of pluripotent cells onto damaged myocardial tissue has not been described in human subjects [100], perhaps due to concerns regarding oncogenicity owing to the vast differentiation potential of PSCs [101]. Numerous factors, such as the generation of heterogeneous cell populations that may include immature cells, concerns about arrhythmogenicity, and immunogenicity due to the expression of different human leukocyte antigen (HLA) markers, seem to be at the root of the difficulties in translating PSC-derived cell studies into the clinic. However, autologous patient-derived iPSCs or HLA-matching of iPSC populations (stem cell banks) may offer a solution, at least in terms of the immunogenicity of administered cell populations. Nonetheless, clinical trials have been or are currently being conducted to study the effects of PSC-derived cells in IHD patients. Two studies have been set up to test direct transplantation of PSC-derived cardiomyocytes [102]; HEAL-CHF has been evaluating the effect of epicardially injected PSC-generated cardiomyocytes on heart failure patients with indications for coronary artery bypass graft (CABG) [89], while LAPiS, a phase I/II clinical trial with an estimated completion date in 2024, will similarly be assessing the effect of iPSC-derived cardiomyocyte spheroids on heart failure patients, more than one month after an episode of MI with a low ventricular ejection fracture (LVEF), through injection [90].

More recently, Muse cells, i.e., cells expressing pluripotency markers, have been isolated and tested; they seem to carry a lower tumorigenic potential themselves since they express low quantities of tumorigenic transcription factors, attributed to increased *let-7 (lethal-7*) expression, which suppresses *Lin28*, a transcription factor contributing to the pluripotent phenotype and, as a result, tumorigenicity. Muse cells also exhibit reduced telomerase activity as well as increased capability for DNA repair [103]. Muse cells have been isolated from various locations, including the bone marrow (BM), connective tissues in various organs, as well as the amniotic membrane; these cells, like PSCs, are capable of differentiation into tissues from all three germ layers [104]. Use of Muse cells in animal models, including intravenous administration in a rabbit model of MI, revealed improvements in ejection fraction (EF), infarct scar size reduction and differentiation into relevant cell types [94]. The mechanism for this effect is thought to be sphingosine monophosphate (S1P)-sphingosine 1 monophosphate receptor 2 (S1PR2) signaling [94], an interaction already observed to facilitate the homing of certain immune and progenitor cells [105]. Similarly promising results were observed in larger animal models when Muse cells were administered in swine models of acute MI; infarct size seemed to be reduced, with associated improvements in ejection fraction and ventricular dimensions and no evidence of arrhythmias [96].

Muse cells have also been utilized in human MI patients in a clinical study commenced in 2018; results showed improved ventricular function and reduced scar size, most likely due to direct cardiomyocyte differentiation as well as local paracrine effects. Nevertheless, only three patients were enrolled in this clinical study and were only followed for 12 weeks; thus, any possible tumorigenicity was not properly assessed [91], even though in general, Muse cells have been described as non-tumorigenic compared to other PSC types [104,106].

In essence, the effects of pluripotent stem cell transplantation may be summarized as effects due to differentiation into local cell populations, observable through the identification of engrafted cells expressing cardiac markers (alpha Myosin heavy chain-aMHC, troponin I-TnI) [88]. Pluripotent cells may also be selected beforehand for their cardiomyogenic potential to better control their differentiation into cardiac cell lineages upon transplantation [88]. Another possible mechanism of action for pluripotent stem cell therapies might include the cardioprotective effects of extracellular vesicles (EV) secreted by PSC-derived cell populations; it seems that PSC-EV administration might not be associated with teratoma formation, as opposed to PSCs themselves, based on results from associated studies [107]. Some authors posit a possible paracrine effect of transplanted pluripotent populations, possibly through the secretion of factors such as cystatin C, osteopontin, clusterin or tissue inhibitor of metalloproteinase-1 (TIMP-1), capable of preventing local cardiomyocyte apoptosis and excess fibrosis. This improves local myocardial tissue viability, in turn enhancing cardiac function [87]. Furthermore, while most studies have shown positive effects after transplantation, some studies do not seem to report long-term engraftment of selected cells; it is thus thought that paracrine effects are most likely the reason for cardiac improvement in the long term [108].

Some drawbacks specifically encountered with pluripotent stem cell populations are immunogenicity and tumorigenicity; although the former may be amended through autologous derivation of iPSC populations, this might extend the therapeutic timeline significantly, mainly due to the time required for iPSC line derivation [108]. Tumorigenicity is another disadvantage frequently encountered with PSCs but not Muse cells; this may be attributed to a variety of factors, including residual, undifferentiated PSCs still capable of growth, genomic instability after multiple cell passages, or even persistent epigenetic modifications long after differentiation into target lineages. To this end, multiple methodologies for evaluating this tumorigenic potential have been created, and although a case-by-case testing basis is feasible, validation studies involving multiple sites have been organized as well [109].

#### 3.1.2. Multipotent Stem Cells in Animal Studies and Clinical Trials

**Figure 2:**
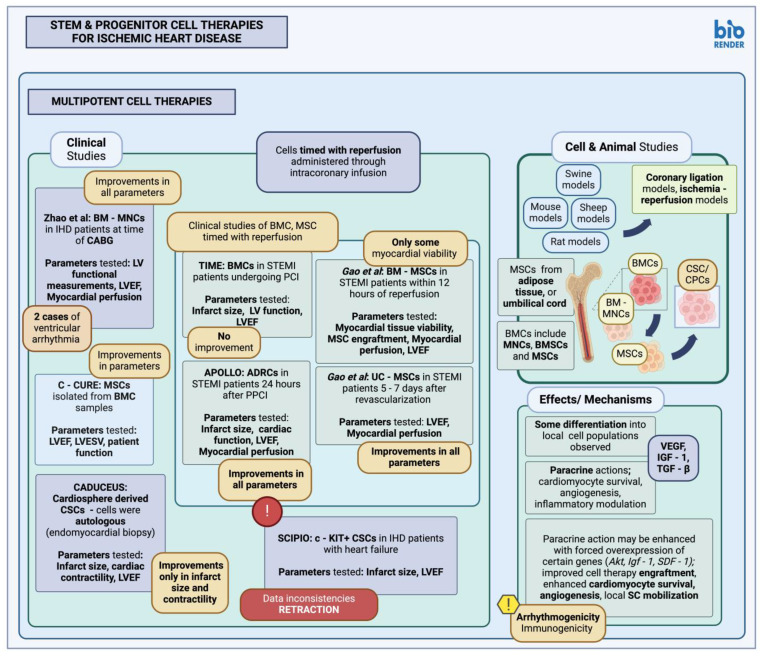
Stem and progenitor cell therapies for ischemic heart disease—multipotent stem cell therapies (created with BioRender.com, accessed on 24 February 2024) [115,116,117,118,122,123,126,127,128]. LV, left ventricle; LVESV, left ventricular end systolic volume; LVEF, left ventricular ejection fraction; MSC, mesenchymal stem cells; CABG, coronary artery bypass graft; STEMI, ST-elevation myocardial infarction; BM, bone marrow; BMC, bone marrow cells; BMSCs, bone marrow stem cells; BM-MNC, bone marrow mononuclear cells; MNC, mononuclear cells; c-KIT, tyrosine protein kinase kit; C-CURE, cardiopoietic stem cell therapy in heart failure; TIME, timing in myocardial infarction evaluation; PCI, percutaneous coronary intervention; PPCI, primary percutaneous coronary intervention; *Akt*, protein kinase B; *Igf-1*; *Insulin-like growth factor 1*; VEGF, vascular endothelial growth factor; *SDF-1*, stromal cell-derived factor 1; TGF-β, transforming growth factor beta; ADRC, adipose-derived regenerative cells; UC-MSC, umbilical cord mesenchymal stem cells; CSC, cardiac stem cell; CPC, cardiac progenitor cell; SCIPIO, stem cell infusion in patients with ischemic cardiomyopathy; CADUCEUS, cardiosphere-derived autologous stem cells to reverse ventricular dysfunction; SC, stem cell.

The use of multipotent stem cells, i.e., cells capable of generating cells relevant to the tissue they are usually found in animal studies and in human patients, has been well described [110]. Examples of multipotent stem cell types used for this purpose include bone-marrow (BM)-derived cells (BMC), mesenchymal stem cells (MSCs) and cardiac stem/progenitor cells (CPC/CSC) (summarized in Table 3, Figure 2). BMCs are perhaps among the earliest used multipotent stem cells for cardiac applications [82]. In fact, the first study reporting their use was published in 2001, where BMC transplantation seemed to cause new cardiac tissue formation 9 days post-implantation [111]. BMCs in general represent a mixed cell population; more specifically, in both small and large animal models, BM-derived mononuclear cells (BM-MNCs) have been used either through intramyocardial injection or intracoronary injection, with promising results and subsequent improvement in ventricular function as well as new local vessel formation, especially in the studies by Kamihata et al. [111,112]. Other studies, however, did not show significant improvement in function or any differentiation of transplanted cells into appropriate local phenotypes [113,114].

**Table 3:** Summary of relevant studies in animals and human patients, utilizing multipotent stem cells, either administered as is, or indirectly, through derivation or relevant, differentiated cell populations.

Study	Cell Type	Model	Method	Comments
Orlic et al., 2001 [111]	BMC	Mouse	Intramyocardial injection	Cells administered post-MI (coronary artery ligation); BMCs were selected based on Lin (Lin-) and c-KIT (c-KIT+) expression. New cardiomyocytes and blood vessels were observed in the infarct border 9 days after administration.
Kamihata et al., 2001 [112]	BM-MNC	Swine	Intramyocardial injection	Cells administered post-MI (LAD ligation); BM-MNCs were shown to contribute to endothelial cell lineages in the area, increasing capillary density, improving local blood flow and cardiac function.
Bel et al., 2003 [113]	BMC	Sheep	Intramyocardial injection	Cells administered post-MI (LCx ligation); no differentiation of implanted BMCs into endothelial cells, or considerable BMC engraftment was observed. The study detected no differences in LV ejection fraction, global wall motion, or LV geometric parameters between control and experimental groups.
de Silva et al., 2008 [114]	BMC	Swine	Intracoronary infusion	Cells administered post-MI (LAD occlusion and reperfusion); no differentiation of implanted cells was observed through subsequent immunofluorescence studies. The study detected no differences in LV ejection fraction, ventricular volume, or size of the resulting infarct between control and experimental groups.
Bartunek et al., 2013 [115]	BM-MSC	Clinical trial	Endomyocardial injection	C-CURE trial; BMC samples selected for MSCs, with subsequent MSCs administered in patients with chronic heart failure due to IHD. From the parameters tested results include an improvement in the LV ejection fraction, reduced LV end-systolic volume, and improvement in patient function.
Gao et al., 2013 [116]	BM-MSC	Clinical trial	Intracoronary infusion	BM-derived MSCs were administered in patients with STEMI undergoing reperfusion within 12 h. The study reported some improvement in cardiac tissue viability, although no significant improvement was observed in terms of MSC engraftment, cardiac tissue perfusion, function (LVEF), between experimental and control groups.
Traverse et al., 2014 [117]	BMC	Clinical trial	Intracoronary infusion	TIME trial; BMCs were administered in patients with STEMI, undergoing PCI, 3 or 7 days after the event. The study reported no significant differences in LV ejections fraction, local LV function, as well as infarct size reduction between the experimental and control groups.
Zhao et al., 2008 [118]	BM-MNC	Clinical trial	Intramyocardial injection	BM-MNCs administered in IHD patients, at time of CABG; the study reported improved LV geometric parameters compared to control (LV wall thickness), improved LV ejection fraction, improved local myocardial perfusion. The study reported two instances of ventricular arrhythmia after BM-MNC administration.
Berry et al., 2006 [119]	MSC	Rat	Intramyocardial injection	Cells administered post-MI (LAD ligation); improvement in cardiac function, and reduced rate of apoptosis and local fibrosis was observed, although there was no effect on local angiogenesis. No differentiation of injected MSCs into differentiated cardiomyocyte was observed.
Gnecchi et al., 2005 [120]	MSC-*Akt* medium	Rat	Intramyocardial injection	Medium conditioned with hypoxic MSC overexpressing *Akt*, was administered post-MI (coronary occlusion); results showed a reduction in infarct size, and cardiomyocyte apoptosis.
Haider et al., 2008 [121]	MSC	Rat	Intramyocardial injection	IGF-1 modified MSCs administered post-MI (coronary artery ligation); MSCs were modified to overexpress *Igf-1*, resulting in higher, local MSC engraftment and survival. There were improvements in LV function parameters and local angiogenesis, as well as reduction in infarct size after administration of MSC-SDF-1+ cells. The study reported increased local stem cell mobilization (CD31+, c-KIT+, MDR1+, CD34+), due to the SDF-1 secretion from transplanted MSCs.
Houtgraaf et al., 2012 [122]	ADRC	Clinical trial	Intracoronary infusion	APOLLO trial; adipose-derived multipotent stem cells were administered in STEMI patients, 24 h after primary PCI. ADRCs in question were patient-derived through liposuction. The study reported improvement in LV ejection fraction and cardiac function overall, reduced infarct size, and improved local myocardial perfusion.
Gao et al., 2015 [123]	UC-MSC	Clinical trial	Intracoronary infusion	Cells administered in STEMI patients 5–7 days after revascularization; the study reported improvements in LV ejection fraction, and myocardial perfusion.
the SCIENCE investigators, 2023 [124]	AD-MSC	Clinical trial	Intramyocardial injection	Cells administered in patients with heart failure due to chronic ischemic heart disease; no significant improvement in LVESV, LVEF, cardiac function.
Bolli et al., 2013 [125]	CSC	Swine	Intracoronary infusion	c-KIT+ CSCs administered post-MI (ischemia-reperfusion); cells expressing sarcomeric proteins, staining positive for Ki67, were identified, along with cells expressing cardiac markers alluding to local generation of differentiated cardiomyocytes. Differentiation into vascular structures was also reported. The study reported increased LV ejection fraction and improved hemodynamic measurements after CSC infusion.
Makkar et al., 2012 [126]	CSC	Clinical trial	Intracoronary infusion	CADUCEUS trial; cardiosphere-derived CPC/CSC were administered in patients. Cells were autologous, procured through endomyocardial biopsy. The study reported, among others, a decrease in scar size, and an increase in cardiac contractility; however, no significant improvement in LV ejection fraction was reported.

MI, myocardial infarction; LV, left ventricle; LVESV, left ventricular end systolic volume; LVEF, Left ventricular ejection fraction; MSC, mesenchymal stem cells; CABG, coronary artery bypass graft; STEMI, ST-elevation myocardial infarction; BM, bone marrow; BMC, bone marrow cells; BM-MNC, bone marrow mononuclear cells; LAD, left anterior descending; LCx, left circumflex artery; Lin, lineage variant; c-KIT, tyrosine protein kinase kit; C-CURE, Cardiopoietic stem Cell therapy in heart failURE (study abbreviation); TIME, timing in myocardial infarction evaluation; IHD, Ischemic heart disease; PCI, percutaneous coronary intervention; Akt, protein kinase B; IGF-1; insulin-like growth factor 1; SDF-1, stromal cell-derived factor 1; CD31, cluster of differentiation 31; CD34, cluster of differentiation 34; MDR1, multidrug-resistant protein 1; ADRC, adipose-derived regenerative cells; UC-MSC, umbilical cord mesenchymal stem cells; CSC, cardiac stem cell; CPC, cardiac progenitor cell; Ki67, marker of proliferation Kiel 67; SCIPIO, stem cell infusion in patients with ischemic cardiomyopathy; CADUCEUS, cardiosphere-derived autologous stem cells to reverse ventricular dysfunction.

Clinical trials utilizing BMCs to try and tackle the cardiomyocyte loss associated with MI have been carried out as well. Some of these studies employed additional measures to select specifically for mesenchymal stem cells (MSC) within the bone marrow sample [115,116], while others used BMCs or BM-MNCs, either through intracoronary administration [117] or through intramyocardial injection along with coronary artery bypass graft (CABG) surgery, respectively [118]. While Bartunek et al. and Zhao et al. reported improvements in geometric and functional parameters after therapy [115], others reported no significant improvement after the employed cell therapy [116,117]. A published meta-analysis has shown that BMC administration may lead to improvement in ventricular function, infarct size, as well as the resulting cardiac remodeling [129], while another one, which evaluated the administration of BMCs specifically at the time of CABG, further exhibited the capacity for BMCs to improve left ventricle (LV) functional parameters when administered intraoperatively [130].

MSCs have also been used for the alleviation of post-MI myocardial tissue injury, MSCs possess abilities for the generation of differentiated connective tissue cell progeny, mitigation, and modulation of the inflammatory response [131]. Various animal studies have been conducted to test the effect of MSC on alleviating MI sequelae. Among them was a study of intramyocardial MSC injection in a rat model, which exhibited a reduction in local, post-infarct fibrosis, improvements in left ventricular wall compliance, and, as a result, better contractility, and function [119]. The improvement in the tested parameters, including infarct size and rate of cardiomyocyte apoptosis, is thought to occur not only through MSC differentiation towards cardiac cell lineages, which some studies did not report at all [119], but mostly through local paracrine effects that may induce angiogenesis or aid in cardiomyocyte protection during hypoxic conditions. In fact, MSCs overexpressing *Akt* were shown to act in a mostly paracrine manner by aiding local cardiomyocyte survival [120], while MSCs overexpressing *Igf-1* aided in the local recruitment of stem and progenitor cells as well. In the latter, the main mechanism for MSC-associated cardiac tissue repair was observed to be activation of the stromal-derived factor-1 (SDF-1)/CXCR4 signaling pathway [120,121,132].

The effect of MSCs on post-MI cardiac tissue structure and function has also been examined in clinical studies. MSCs have been isolated from the BM, adipose tissue (AT) (AT-derived multipotent stem cells), and the umbilical cord (UC), with no difference in isolation rate, multipotency characteristics or immune characteristics between the different MSC types [132,133]. The APOLLO trial, which involved administration of MSC-like cells derived from adipose tissue, denoted as adipose tissue-derived regenerative cells (ADRC), through intracoronary injection 24 h after primary PCI revascularization. The study reported promising results, with regard to scar size reduction and overall cardiac function [122]. However, similar trials utilizing adipose-derived MSCs did not report any significant improvements in cardiac function [124]. Another trial utilized umbilical cord-derived MSCs (UC-MSC) administered through intracoronary infusion in patients with STEMI, five to seven days after revascularization; the results in these studies were promising here as well, with improvements in LV function and myocardial perfusion [123,132].

CSC/CPCs include many different cell types, with many also possessing the capacity for generating differentiated cardiomyocytes, as detailed previously. Procurement may be autologous [101,134]; however, this may prove cumbersome due to the limited number of such cells within cardiac tissue [135]. CSC/CPCs thus far have been used in various animal studies, including c-KIT+ cells introduced through intracoronary infusion in a swine model of MI. In this study, implanted cells exhibited differentiation into both cardiac and vascular phenotypes and led to improved left ventricular function [125,136]. In general, implanted c-KIT+ cells show better capacity for scar reduction compared to MSCs [136,137]. Other studies have also utilized cardiosphere-derived progenitors through intracoronary injection, with similarly promising results [136].

CSCs/CPCs have also been used in clinical trials. Two relevant clinical trials have utilized autologous c-KIT+ and cardiosphere-derived cells, namely the SCIPIO and CADUCEUS trials, respectively [137]. In the SCIPIO trial autologous c-KIT+ cardiac progenitors were administered through intracoronary injection; the study was later retracted in 2019 [127,128,138]. Another similar trial, the CADUCEUS trial, examined the effect of cardiosphere-derived cells, administered through intracoronary injection, on infarcted hearts; results included an observed reduction in scar size, although this trial reported no effect on left ventricular ejection fraction [126].

The action of multipotent stem cell populations mostly lies in their ability to produce appropriate local factors that might aid in local cardiomyocyte survival and apoptosis, angiogenesis, or influence inflammatory pathways; however, they may also exhibit some capability for differentiation into cardiomyocyte-like cells, although the functionality of this progeny may not always be as clear-cut, as is the case with adipose-derived stem cells, for example [139]. As for secretory activity, this may include factors such as vascular endothelial growth factor (VEGF), insulin-like growth factor 1 (IGF-1) or transforming growth factor β (TGF-β) [140]. A multitude of different multipotent stem cell populations have been utilized in a multitude of different animal studies and clinical trials, including BM-derived populations, MSCs derived from various locations, as well as CSC/CPC. Results have been variable, although in cases of successful studies, the main effects have been improvements in local perfusion and capillary density, myocardial tissue viability and cardiac function, as well as infarct size [141]. This secretory function may be augmented through forced overexpression of certain genes, including *Igf-1*, *Akt* and *stromal derived growth factor-1* (*Sdf-1*); when overexpressed, these may further facilitate the local function of these stem cell therapies or aid in recruitment of local stem and progenitor cells, further aiding in cardiomyocyte survival, angiogenesis, and overall improvement in cardiac function. Paracrine secretion also seems to be the mechanism of action for CPC/CSC populations as well, as further proven by studies that do not seem to assign any role for differentiated cardiomyocyte generation by c-KIT+ and SCA-1+ cells [142]. Additional mechanisms in these cases seem to be activation of the SDF-1/CXCR4 pathway, at least in cases of IGF-1 overexpression [120,121]. In general, however, studies employing multipotent stem cell populations seem to exhibit variable results, mostly due to variation in the stem cell type administered, the dose, the method of administration, or any additional biological alterations that may improve their efficacy; this might also serve as an explanation for the lack of a unified mechanism of action across different studies since different therapeutic approaches employ different mechanisms [143].

Although not tumorigenic or immunogenic when derived from autologous sources or when immunomodulatory MSCs are specifically utilized, as evident from relevant studies where xenogeneic MSCs do not seem to trigger a significant immune reaction [144], administration of multipotent stem cells, as with pluripotent stem cells, may also result in arrhythmias (arrhythmogenicity). Various mechanisms may contribute to this observed arrhythmogenicity; one such mechanism is pacemaker-like activity, or depolarizations from implanted cells [145], as well as negative effects on cellular excitation of implanted cells due to secretion of local factors, frequently observed after MSC transplantation [146]. Another mechanism may be tissue heterogeneity due to the innate variability of cellular excitation in the cellular sample introduced, oftentimes owing to differences in their electrophysiological profile; this may include variations in ion currents or even differential expression of ion channel or gap junction proteins. Finally, increased cellular automaticity, exacerbated by the high proportion of fibroblasts within the infarct scar, might also play a role [142].

### 3.2. Tissue-Engineered Therapies

Stem cells may be delivered to the target area of post-MI ischemia, fibrosis, or necrosis in the form of a tissue-engineered (TE) construct. This may be in the form of a scaffold-derived product, generated from compatible biomaterials and infused with the appropriate cell types. These so-called myocardial patches may then be applied directly to the cardiac surface, either as monotherapy or combined with surgical revascularization. TE therapies for the treatment of post-MI cardiac tissue injury may also be delivered in the form of a cell sheet construct. In any case, delivery of appropriate cell types in a more rigid medium, compared to direct cell infusion, might address issues with cell engraftment and retention in the area, a problem frequently encountered with cell therapies [147,148].

#### 3.2.1. Pluripotent Stem Cell Constructs 

**Figure 3:**
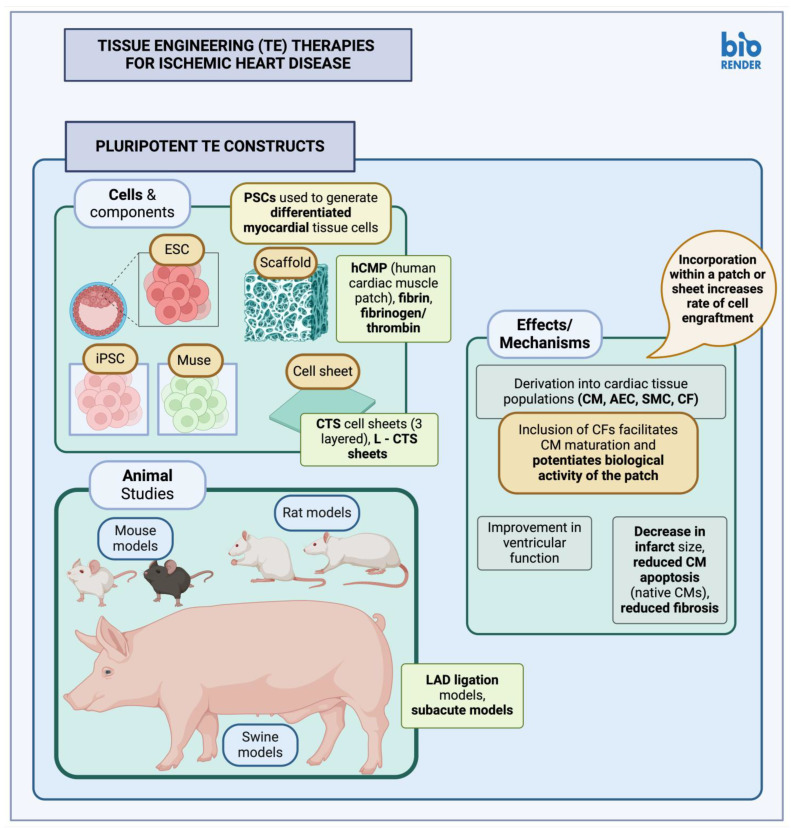
Tissue engineering (TE) therapies for ischemic heart disease—Pluripotent TE constructs (created with BioRender.com, accessed on 24 February 2024). TE, tissue engineering; ESC, embryonic stem cells; iPSC, induced pluripotent stem cells; PSC, pluripotent stem cells; CTS, cardiac tissue sheet; L-CTS, large cardiac tissue sheet; CM, cardiomyocyte; LAD, left anterior descending coronary artery; hCMP, human cardiac muscle patch; hPSC, human pluripotent stem cell; hESC, human embryonic stem cell; SMC, smooth muscle cells; AEC, arterial endothelial cells; CF, cardiac fibroblast.

Human-derived iPSCs (hiPSCs) have been used to generate cell sheet in both small and large animal models of MI (Table 4, Figure 3). iPSCs in these cases were used to derive tissue-specific cells and progenitors, although in some instances a mix of iPSCs and embryonic stem cells (ESC), collectively referred to as PSCs, have been utilized, as in the study by Lou et al. [149]. Amount of cell retention, new vessel formation, and alterations or improvements in myocardial function were some of the common parameters tested in most of these studies. Results in general seem to be positive [150,151,152], though cell engraftment observed in some swine models seems to be diminished [151,153]. iPSCs have the developmental potential for the generation of many different cell lineages; during iPSC reprogramming, however, some epigenetic modifications might persist in the starter cell populations. It has thus been proposed that it might be more favorable to use cardiac lineages for iPSC derivation instead [152].

PSC-derived cells may also be delivered to target tissues within fibrin scaffolds (Table 5); the addition of fibroblasts along with other commonly delivered, differentiated cell types may further enhance local tissue recovery. The addition of cardiac fibroblasts, for example, along with other cardiac-tissue-specific cell types, seems to enhance the maturation of cardiomyocytes within the patch, which is thought to occur through the enhanced intracellular cAMP signaling pathway, as well as further aid in local tissue recovery due to improvements in the rate of cardiomyocyte engraftment [149]. The observed positive effects may also be due to altered mechanical properties imparted by cardiac fibroblasts, or more specifically, due to their secretion of extracellular matrix (ECM) [149]. Myocardial patches of larger size have also been created and applied to larger animal models. In these cases, the results have been promising and may perhaps facilitate the translation of similar studies into a clinical setting [149,154].

#### 3.2.2. Multipotent Stem Cell Constructs

**Figure 4:**
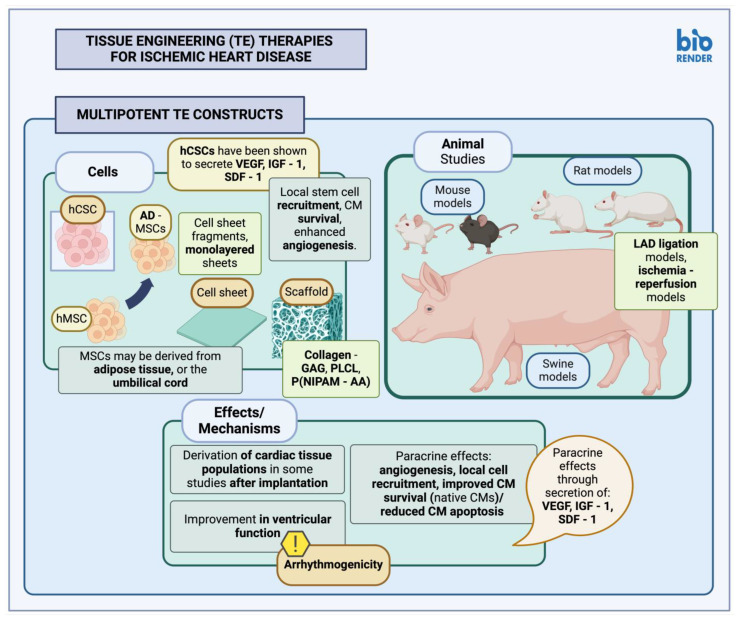
Tissue engineering (TE) therapies for ischemic heart disease—Multipotent TE constructs (created with BioRender.com, accessed on 24 February 2024). TE, tissue engineering; CM, cardiomyocyte; LAD, left anterior descending coronary artery; MSC, mesenchymal stem cells; hCSC, human cardiac stem cells; hMSC, human mesenchymal stem cells; AD-MSC, adipose-derived mesenchymal stem cells; GAG, glycosaminoglycans; PLCL, poly(lactide-co-ε-caprolactone); P(NIPAM-AA), poly(N-isopropylacrylamine-co-acrylic acid); VEGF, vascular endothelial growth factor; IGF-1, insulin-like growth factor 1; SDF-1, stromal cell-derived factor-1.

Myocardial patches utilizing multipotent cells derived from the bone marrow (BM) or adipose tissue (AT) have also been generated (Table 5, Figure 4); these have been applied in both small and larger animal models, suspended in various types of polymeric materials, including type I collagen [155,156], PLCL (poly(lactide-co-ε-caprolactone) [157], and poly(lactic-co-glycolic acid) [159], or generated as scaffold-free cell sheets [158], or alternatively, cell sheet fragments [160]. Cells may also be encapsulated in materials aiding in their survival when transplanted onto the target tissues; Tang et al., for example, have utilized a thermosensitive nanogel composed of P(NIPAM-AA) (poly(N-isopropylacrylamine-co-acrylic acid)), which has been shown to facilitate the favorable actions of multipotent stem cells when applied in both small and larger animal models [161].

Most of these constructs have generally led to improvements in cardiac function in relevant experiments, as well as local angiogenesis. In other studies, however, positive results were thought to be due to increased local generation of myofibroblasts [156]. Delivery of stem cells in a biomaterial matrix seems to aid in local cell survival since infarcted myocardial tissue is thought to be a poor environment for proper cellular growth and differentiation [157]. In addition, MSC administration seems to be favorable for inducing local biological processes aiding in tissue repair, including angiogenesis [158]. Some studies, however, reported arrhythmias after graft implantation [160]. It is also interesting to note that while pluripotent cells have been used indirectly in most studies, in order to derive relevant cell progeny suitable for cardiac implantation, multipotent cell constructs such as MSCs have also been administered as is, perhaps due to both their differentiation potential and their favorable paracrine effects [153]. Additional multipotent cell types used in similar studies include CPC/CSCs; these cells, although theoretically more inclined towards a cardiac differentiation lineage, have also been shown to mainly act through local effects, including secretion of proteins such as VEGF, IGF-1 and SDF-1, facilitating local angiogenesis, recruitment of endogenous stem cell lineages, and preventing marked cardiomyocyte apoptosis, thus enhancing overall local cellular survival [161,162].

Translation of preclinical studies involving TE constructs along with stem cells, or stem cell derivatives to a more clinical application seems to be difficult. There seem to be various issues arising due to inappropriate adhesion to underlying tissues (either not enough or inappropriately increased adhesion) and immunogenicity, not only of cellular products but of scaffold material as well. Arrhythmogenicity also seems to be a problem in some studies, which might require further research before a clinical application [162].

## 4. Conclusions 

Despite recent advancements, it seems conventional therapeutic strategies for MI are still lacking or may be associated with side effects/complications of varying intensity. Cardiac surgery and other interventional methods, though necessary, can be associated with morbidity and mortality, as well as a risk for repeat procedures. However, progress in stem cell research, including new stem cell therapies as well as the use of biomaterials and tissue engineering principles, has spurred a multitude of experimental and clinical studies to evaluate the effect of these factors on myocardial tissue injury post-MI, as well as their potential for inducing or facilitating mechanisms for endogenous tissue regeneration. This is because endogenous regeneration of the myocardium, although indeed occurring at a low rate, is not quite enough to fully aid in mitigation or local regeneration after injury, at least to a clinically relevant level. Thus, additional therapies that might better spur these processes could be a useful adjunct to the various revascularization procedures, including cardiac surgery and PCI.

PSCs have been used as is in experimental studies, as well as indirectly, to derive appropriately differentiated progeny; for clinical trials, however, PSCs have been mostly used indirectly to derive cardiomyocytes, which are appropriate for transplantation. Dangers associated with the use of PSCs, namely tumorigenesis, may be associated both with direct PSC transplantation as well as transplantation of PSC-derived cells; the latter is usually due to residual undifferentiated cells within the sample administered and makes clinical translation of such therapies difficult. A solution for this problem has been sought with the use of Muse cells, pluripotent cells isolated from connective tissues with reportedly low tumorigenic potential. While animal studies with Muse cells have been conducted, more clinical studies with a larger sample size and longer follow-up periods are required to fully evaluate the potential of these cells, including their potential for tumorigenesis. On the other hand, studies evaluating the effect of multipotent cell populations have been numerous, in both animal models and clinical trials; while some have reported no significant effect, particularly studies incorporating BMCs, others have successfully facilitated improvement in cardiac function as well as local myocardial tissue improvement. However, some clinical trials involving multipotent stem cell populations have been marred by controversy, highlighting the need for appropriate reporting of laboratory data.

There seem to have been many different studies conducted evaluating the effect of stem cells on ischemic myocardial tissue, with variable result. Though some possible mechanisms for the action of these therapies have been proposed, a common mode of action has not yet been specifically defined. Furthermore, many of these studies vary in terms of type of cell, cell dose, frequency of administration, and results reported; this apparent lack of “standardization” makes it difficult to adequately compare between different studies, at least with regard to the significance of the therapeutic effect. It would thus be of benefit to create a standardized system for the clinical evaluation of stem cell therapies to better facilitate comparison between different studies and therapies.

A multitude of different signaling mechanisms have been shown to control cardiomyocyte proliferation both during embryonic development as well as adulthood; it would be thus quite interesting to ascertain in additional studies whether forced overexpression of factors that activate cardiomyocyte proliferation might potentiate any positive effect or whether combinatorial overexpression of certain growth factors in target stem cell therapies might potentiate any positive effect. However, care must be taken to prevent overt cellular proliferation after engraftment in such cases, something that may be avoided through the introduction of genetic self-destructive switches, induced on demand, after the introduction of a specific activating substance. The use of such systems may also be useful in curbing the associated tumorigenicity of pluripotent stem cells, helping to improve their safety profile.

Stem cell therapies may be administered in a variety of methods, although intracoronary and direct intramyocardial injections were mostly presented in this review. These methods are most likely to be implemented along with interventional or surgical therapies. Even with direct injection, however, cell retention and engraftment may still be an issue; in this case, tissue engineering methods, through the use of appropriate biomaterials, might aid in better retention and engraftment or even increase the potency of the associated therapy. While many studies have been carried out with animal models, clinical translation seems to still be in its infancy; perhaps more studies utilizing large animal models could better aid in evaluating large, clinically relevant myocardial patches or cell-sheet constructs, better suitable for clinical applications.

Stem cell therapies, especially when combined with tissue engineering methods, offer exciting new alternatives for many different clinical problems. It is with rigorous testing and appropriate evaluation of any problems that may arise that the translation of these therapies into a clinical setting might be more successful.

## Figures and Tables

**Table 1:** Summary of pathophysiological alterations implicated in the development of ischemic heart disease.

Process/ Mechanism	Key Mediators	Comments/Observations
Atherosclerosis	oxLDL, macrophages, VSMCs	Endothelial dysfunction, intimal lipoprotein accumulation, foam cells PLT adhesion, release of TGF-β, FGF VSMC proliferation, ECM deposition (collagen) and fibrous plaque formation. Inflammatory cell infiltrate, extracellular lipid core necrosis and fibromuscular cap composition all influence plaque stability.
Inflammation	oxLDL, macrophages	Endothelial oxLDL adhesion through LOX-1, expression of NF-kB and endothelial adhesion molecules MI: immunogenic CM fragments, upregulation of NF-kB, production of pro-inflammatory cytokines (TNF-α, IL-1β, IL-6, IL-18).
Coronary vessel reactivity	ROCK, PKC	Increased MLC sensitivity to Ca^2+^; direct effect of ROCK on MLC, indirectly effect through ROCK, PKC-mediated, MLCPh inhibition.
Microvascular dysfunction	AGE, ROS, RNS, DAG-PKC	Hyperglycemia: AGEs, ROS impair antioxidant system function and decrease NOS bioavailability (endothelial dysfunction). Intercellular junction disruption through DAG-PKC signaling. Aging: Increased ROS, RNS production

oxLDL, oxidized low-density lipoprotein; PLT, platelet; TGF-β, Transforming growth factor β; FGF, fibroblast growth factor; VSMC, vascular smooth muscle cell; ECM, extracellular matrix; LOX-1, lectin-like oxidized low-density lipoprotein receptor 1; NF-kB, nuclear factor kappa B; MI, myocardial infarction; CM, cardiomyocytes; MLC, myosin light chain; ROCK, Rho kinase; MLCPh, myosin light chain phosphatase; PKC, protein kinase C; AGE, advanced glycosylated end-products; ROS, reactive oxygen species; RNS, reactive nitrogen species; DAG, diacylglycerol; IL-1β, interleukin-1β; TNF-α, tumor necrosis factor alpha; IL-6, interleukin 6; IL-18, interleukin-18.

**Table 2:** Summary of relevant studies in animals and human patients, utilizing pluripotent stem cells (PSCs), either administered as is or indirectly through derivation of relevant differentiated cell populations.

Study	Model	Method	Comments
Singla et al., 2007 [87]	Mouse	Intramyocardial injection	mESCs administered post-MI (LCA ligation); results in the parameters studied: decreased CM apoptosis (TUNEL staining), decreased fibrosis, reduced CM hypertrophy, possibly due to paracrine secretion of factors such as cystatin C, osteopontin, clusterin, TIMP-1. Higher proportion of viable myocardium in the peri-infarct area, in subjects receiving the mESCs.
Kannappan et al., 2019 [92]	Mouse	Intramyocardial injection	iPSC-CM administered post-MI; Nutlin-3a (MDM2 inhibitor) to activate p53, inducing apoptosis in populations, to select for DNA-damage-free iPSCs, before CM derivation. Increased engraftment rate for DNA-damage-free CMs, compared to control CMs.
Min et al., 2003 [88]	Rat	Intramyocardial injection	mESCs administered 20 min post-MI (LCA ligation); mESC populations were selected for cardiomyogenic potential and transfected with GFP to identify engrafted cells. Reduced infarct size, reduced LV mass and LV mass/body weight ratio, mESC engraftment, increased angiogenesis observed through increased capillary density (up to 32 weeks later).
Thavapalachandran et al., 2021 [93]	Rat	Intramyocardial injection	iPSC-MSC administered post-MI; improved LV function, enhanced angiogenesis, no effect on infarct size, no continuous engraftment. No observable arrhythmias (absence of locally generated re-entrant circuits normally produced by sustained iPSC-MSC engraftment causing scar heterogeneity).
Yamada et al., 2018 [94]	Rabbit	Intravenous infusion	Muse cells administered post-MI (ischemia-reperfusion model); results include reduced infarct size, increased ejection fraction, reduced left ventricle geometric values, with engrafted Muse cells identified in the target tissues (up to 6 months later). Muse migration towards target tissues through S1P-S1PR signaling.
Chong et al., 2014 [95]	Pigtail macaque	Intramyocardial injection	hESCs-CMs administered post-MI (percutaneous ischemia-reperfusion injury model); reduced infarct size, remuscularization, appropriate revascularization in the infarcted area, no statistically significant effect on LV function. Arrhythmias observed in subjects receiving hESC-CMs; PVC, wide QRS rhythms, ventricular tachycardia; this is contrast to previously similar experiments in small animal models.
Yamada et al., 2022 [96]	Swine	Intravenous infusion	Muse cells administered post-MI (ischemia-reperfusion model); results include reduced infarct size, increased ejection fraction, reduced left ventricular geometric values. No arrhythmias were observed during the study.
Help Therapeutics, 2022 [89]	Phase I/II clinical trial	Epicardial injection	HEAL-CHF trial; hPSC-CMs administered at the time of CABG. Parameters measured include sustained ventricular arrhythmias, tumorigenesis, infarct size, ventricular wall thickness, MACE (death, non-lethal MI, hospitalization due to the worsening of heart failure) No results posted yet on clinicaltrials.gov (accessed on 25 December 2023)
Heartseed Inc., 2022 [90]	Phase I/II clinical trial	Injection	LAPiS trial; iPSC-CM administered in patients with heart failure, secondary to ischemic heart disease; parameters measured include safety/tolerability, LV ejection fraction, index of myocardial strain, myocardial blood flow and viability, among others. No results posted yet on clinicaltrials.gov (accessed on 25 December 2023)
Noda et al., 2020 [91]	Clinical trial	Intravenous infusion	Muse cells administered post-STEMI, after PCI; only 3 patients were enrolled in the study. Resulting parameters include reduced infarct size, improved LV function and remodeling. No fatal arrhythmias reported for the duration of 12 weeks after therapy administration.

mESCs, mouse embryonic stem cells; MI, myocardial infarction; LCA, left coronary artery; CM, cardiomyocyte; TUNEL, terminal deoxynucleotidyl transferase biotin-dUTP (uridine 5′ triphosphate) nick end labeling; TIMP-1, tissue inhibitor of metalloproteinase 1; GFP, green fluorescent protein; LV, left ventricle; iPSC, induced pluripotent stem cells; MSC, mesenchymal stem cells; MDM2, murine double minute 2; p53, tumor protein 53; DNA, deoxyribonucleic acid; hESCs, human embryonic stem cells; PVC, premature ventricular contractions; CHF, congestive heart failure; hPSC, human pluripotent stem cells; CABG, coronary artery bypass graft; MACE, major adverse cardiovascular events; S1P, sphingosine-1-phosphate; S1PR, sphingosine-1-phosphate receptor; STEMI, ST-elevation myocardial infarction.

**Table 4:** Summary of relevant studies in animals and human trials, utilizing pluripotent stem cells, to derive appropriate tissue-engineered (TE) constructs.

Study	Cell Type	Model	Constructs	Comments
Masumoto et al., 2014 [150]	hiPSC	Rat	hiPSC-CTS cell sheet	hiPSC used to induce cardiac and vascular tissue cells simultaneously (CMs, ECs, vascular MCs) through protocols utilizing Dkk1, VEGF and an intermediate mesodermal linage stage. A three-layered cell sheet was transplanted post-MI (subacute model); ventricular wall contraction was improved, along with other cardiac function parameters, post-MI myocardial fibrosis was reduced. Cell engraftment was also successful, with relevant area of engraftment up to 44%; there were also clusters of ECs around CMs, alluding to the possibility for local angiogenesis, although a vascular structure of graft origin could not be verified.
Ishigami et al., 2018 [151]	hiPSC	Swine	L-CTS sheet	Cardiac tissue populations (CMs, ECs, vascular MCs) were induced from human iPSC populations; cell sheets of clinical-grade size (L-CTS) were created. L-CTS were transplanted post-MI (LAD ligation); results showed improved systolic function and LV ejection fraction, decreased local fibrosis, improved capillary density.
Zhang et al., 2015 [152]	hciPSC	Mouse	CM sheet	hciPSCs were generated from human left atrial appendage tissue and differentiated into CMs; CM cell sheets were created with the Matrigel sandwich method. CM sheets were implanted post-MI (LAD ligation); results showed improvements in cardiac function, increased local vascularity, and reduced native CM apoptosis.
Lou et al., 2023 [149]	hESC, hiPSC	Mouse	hCMP	hPSCs were used to derive hPSC-CMs, hPSC-SMCs, hPSC-AECs, hPSC-CFs, which were then suspended in fibrinogen and thrombin within an appropriately shaped mold, to create hCMPs. Inclusion of cardiac fibroblasts aided in CM maturation (sarcomere structure and organization, CM potentials), within hCMPs, as well as hCMP engraftment. hCMPs were implanted post-MI (LAD ligation); results showed improved LV ejection fraction, decreased infarct size.
Gao et al., 2018 [154]	hiPSC	Swine	hCMP	hiPSCs were used to generate CM, EC, SMC, which were then suspended within fibrin on an appropriate scaffold, to generate hCMPs. In this study, hCMPs constructed were large and thick enough to test a clinically relevant product. hCMPs were implanted post-MI (LAD ligation); the study showed improved LV function, reduced infarct size, a decrease in associated myocardial hypertrophy as well as reduced apoptosis of native CMs.

hiPSC, human-induced pluripotent stem cells; CTS, cardiac tissue sheet; L-CTS, large cardiac tissue sheet; CM, cardiomyocyte; EC, endothelial cell; MC, mural cell; Dkk1, Dickkopf wingless/integrated (WNT) signaling pathway inhibitor 1; VEGF, vascular endothelial growth factor; MI, myocardial infarction; hciPSC, human cardiac induced pluripotent stem cell; LAD, left anterior descending coronary artery; hCMP, human cardiac muscle patch; hPSC, human pluripotent stem cell; hESC, human embryonic stem cell; SMC, smooth muscle cells; AEC, arterial endothelial cells; CF, cardiac fibroblast; LV, left ventricle.

**Table 5:** Summary of relevant studies in animals and human trials, utilizing multipotent stem cells, to derive appropriate tissue-engineered (TE) constructs.

Study	Cell Type	Model	Constructs	Comments
Xiang et al., 2006 [155]	MSC	Rat	Collagen-GAG scaffold	Construct administered post-MI (LAD ischemia reperfusion); the study evaluated effect of ECM cross-linking methods in contrast efficacy. Only constructs generated through DHT crosslinking and subsequent carbodiimide treatment seemed to survive engraftment, although MSCs within both scaffold types seemed to survive. Results showed improved angiogenesis in both scaffold groups.
Simpson et al., 2007 [156]	hMSCs	Rat	Cardiac patch	Patches composed of a rat tail type I collagen scaffold were administered post-MI (LAD ligation); although successful MSC engraftment was reported, there were no detectable hMSC populations after 4 weeks, despite persistence of improvements. The study reported improvements in cardiac geometric parameters and hemodynamic measurements; mechanism for these effects was thought to be increased local myofibroblasts (a-SMA+), both patch-derived as well as due to local recruitment.
Jin et al., 2009 [157]	MSC	Rat	PLCL	Constructs were administered post-MI (cryoinjury method); evidence of myogenesis was observed in injured tissue after implantation, denoted through an increase in cardiac markers (MHC, a-actin, Troponin I), as well as through detection of labeled MSCs expressing a-actin, Troponin I post-implantation (higher in the groups that received MSCs via scaffold) Results showed decreased area of infarct, improved cardiac function and LV ejection fraction.
Miyahara et al., 2006 [158]	AD-MSC	Rat	Monolayered MSC sheet	Cell sheets implanted post-MI (coronary ligation); cell sheets appeared thickened post-implantation, with evidence of cardiomyocyte differentiation, and angiogenesis. The study reported improved cardiac function, and reversal of myocardial wall thinning after MI.
Zhang et al., 2023 [159]	AD-MSC	Rat	PLGA scaffold	Construct administered post-MI (LAD ligation); histological evaluation including fibrosis, angiogenesis, and cardiac remodeling, along with echocardiography. Reduced cardiac fibrosis, cardiac hypertrophy observed post-injury, increased new blood vessel formation in the infarct border zone, functional improvement.
Huang et al., 2013 [160]	MSC	Swine	MSC cell sheet fragments	Cell sheet fragments generated through thermo-responsive methylcellulose hydrogel system; lack of proteolytic enzymes during generation to prevent dissociation of MSCs from their surrounding ECM. MSCs used in the study were autologous. Mechanism of action was thought to be MSC differentiation into relevant cell types (endothelial cells, smooth muscle cells), lack of MSC dissociation from their associated ECM. Cell sheet fragments implanted post-MI; results showed improved cardiac function, and infarct size. The study reported arrhythmias associated with MSC implantation.
Tang et al., 2017 [161]	hCSC	Mouse, Swine	Thermosensitive nanogel	hCSC encapsulation within a nanogel composed of P(NIPAM-AA), which contained hydrophilic moieties, to facilitate hCSC survival and growth; implantation post-MI (LAD ligation), two types of animal models. Results of the study included improved local cardiomyocyte survival, enhanced local angiogenesis, reduced myocardial fibrosis, and improved cardiac function. Mechanism of action was thought to be use of an appropriate hydrogel for cell delivery and survival, and hCSC-mediated secretion of VEGF, IGF-1, SDF-1, facilitating local stem cell recruitment, local cardiomyocyte survival and local angiogenesis.

MSC, mesenchymal stem cells; GAG, glycosaminoglycans; MI, myocardial infarction; LAD, left anterior descending coronary artery; ECM, extracellular matrix; DHT, dehydrothermal treatment; hMSC, human mesenchymal stem cells; a-SMA, alpha smooth muscle actin; PLCL, poly(lactide-co-ε-caprolactone); MHC, myosin heavy chain; LV, left ventricle; AD, adipose-derived; hCSC, human cardiac stem cells; P(NIPAM-AA), poly(N-isopropylacrylamine-co-acrylic acid); VEGF, vascular endothelial growth factor; IGF-1, insulin-like growth factor 1; SDF-1, stromal cell-derived factor-1.
